# PostFocus: automated selective post-acquisition high-throughput focus restoration using diffusion model for label-free time-lapse microscopy

**DOI:** 10.1093/bioinformatics/btae467

**Published:** 2024-07-23

**Authors:** Kwan-Ling Wu, Melisa J Montalvo, Prashant S Menon, Badrinath Roysam, Navin Varadarajan

**Affiliations:** William A. Brookshire Department of Chemical and Biomolecular Engineering, University of Houston, Houston, TX 77204, United States; William A. Brookshire Department of Chemical and Biomolecular Engineering, University of Houston, Houston, TX 77204, United States; William A. Brookshire Department of Chemical and Biomolecular Engineering, University of Houston, Houston, TX 77204, United States; Department of Electrical and Computer Engineering, University of Houston, Houston, TX 77204, United States; William A. Brookshire Department of Chemical and Biomolecular Engineering, University of Houston, Houston, TX 77204, United States

## Abstract

**Motivation:**

High-throughput time-lapse imaging is a fundamental tool for efficient living cell profiling at single-cell resolution. Label-free phase-contrast video microscopy enables noninvasive, nontoxic, and long-term imaging. The tradeoff between speed and throughput, however, implies that despite the state-of-the-art autofocusing algorithms, out-of-focus cells are unavoidable due to the migratory nature of immune cells (velocities >10 μm/min). Here, we propose PostFocus to (i) identify out-of-focus images within time-lapse sequences with a classifier, and (ii) deploy a de-noising diffusion probabilistic model to yield reliable in-focus images.

**Results:**

De-noising diffusion probabilistic model outperformed deep discriminative models with a superior performance on the whole image and around cell boundaries. In addition, PostFocus improves the accuracy of image analysis (cell and contact detection) and the yield of usable videos.

**Availability and implementation:**

Open-source code and sample data are available at: https://github.com/kwu14victor/PostFocus.

## 1 Introduction

High-throughput (HT) assays are pivotal in cell-based drug discovery as they can predict clinical responses, efficacy, and safety. Recent advances in imaging technology have made imaging-based assays an attractive option, as the abundance of content in acquired images makes multiplexed profiling possible (Isherwood *et al.* 2011, Chandrasekaran *et al.* 2021). For example, imaging modalities like phase contrast microscopy provide more morphological information than traditional bright-field microscopy without invasive procedures or synthetic fluorochromes (Pluta 1993), which is beneficial for live-cell imaging. Moreover, breakthroughs in vision algorithms have complemented the profiling workflow with automated image processing and analysis modules.

The integration of these technologies has allowed high-efficiency live-cell profiling and is poised to contribute to advances in cell-based therapeutics. Advancements in synthetic biology (Chakravarti and Wong 2015, Mount *et al.* 2015) and immunotherapies (Oiseth and Aziz 2017, Esfahani *et al.* 2020) with genetically modified lymphocytes have revolutionized the treatment for numerous kinds of cancers, garnering long-term efficacy and approval from the US Food and Drug Administration (Le *et al.* 2018, O’Leary *et al.* 2019, Abramson *et al.* 2020, Fowler *et al.* 2022, Martin *et al.* 2023). Unlike conventional drugs, this new class of living drugs changes their behavior and function dynamically upon encountering tumor cells. Consequently, HT dynamic imaging assays are necessary to map the therapeutic potential of these effector cells. Despite advances in instrumentation and auto-focusing techniques, HT imaging is limited by tradeoffs between imaging frequency and imaging throughput. In other words, when imaging numerous living cells, especially motile cells like immune cells, one of the major challenges is intermittent out-of-focus (OOF) events (Wang *et al.* 2019b).

**Figure 1.**
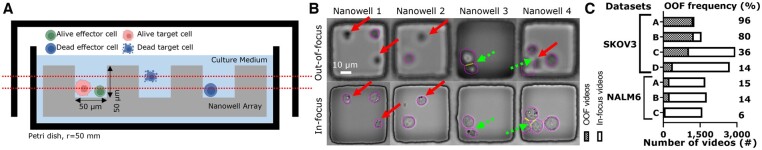
OOF frames can severely reduce the yield of usable videos in HT time-lapse phase contrast microscopy. (A) In the TIMING assay, cells are co-cultured in nanowells (50 μm edge length). As illustrated in the schematic, finding the single best focal plane (dotted lines) for a nanowell is often challenging due to the migratory nature of the cells. (B) Examples of cell-segmentation and cell-contact detection errors that arise for OOF images. The lack of fine features in OOF images results in segmentation errors (darker color as cell contour and brighter color as contour of contact region), which can erroneously indicate missing cells (solid arrows) and false contact events (dashed arrows). (C) Quantifying the loss of usable nanowell videos for seven representative TIMING datasets. The shaded region (left) indicates the number of time-lapse videos of nanowells with more than 5% OOF frames (determined by the classifier described in Fig. 3). The blank region (right) stands for in-focus videos. The number next to each bar indicates the percentage of OOF videos.

OOF objects present a critical challenge for image analysis workflow because a nonoptimal image plane for the camera will introduce a diffusive pattern called OOF blur, leading to low contrast and loss of vision features like edges or corners (Wang *et al.* 2019b, Chen *et al.* 2021). This kind of blur introduces errors in image analysis like missed detection/segmentation of cells or incorrect identification of cell–cell contact (Fig. 1B; Dodge and Karam 2016). This problem is magnified in time-lapse microscopy wherein the construction of the final output depends on processing individual frames and combining these results into temporal sequences. In these videos, even two consecutive OOF frames result in the need to discard the entire video and lead to dramatically lower numbers of usable videos (Fig. 1C). Hence, the scale of OOF videos indicates a potential loss of up to thousands of videos, rendering post-acquisition image focus restoration an essential tool to recover usable data.

The traditional image restoration methods recover in-focus images by deconvolution with known or estimated blur kernels, also known as the point spread function (PSF; Richardson 1972, Wang and Tao 2014). Considering the restrictiveness of applying a spatially uniform kernel, an accurate and comprehensive estimation of PSF is necessary. However, such computation relies on expensive computational optimization that tends to be resource-intensive and time-consuming due to the high number of unknown variables contributing to PSF and the various types of image blurs.

Fortunately, recent developments in deep learning models and training strategies (Martínez-Martínez and Nabavi 2023) have enabled efficient focus restoration by modeling the inverse of the PSF through parameters learned through data-driven approaches (Zhang *et al.* 2019a, Tsai *et al.* 2022, Wang and Han 2022, Wang *et al.* 2022, Yae and Ikehara 2023). In addition to traditional discriminative model-based methods, the de-noising diffusion probabilistic models (DDPMs; Ho *et al.* 2020) have recently succeeded in complicated computer vision tasks like image restoration and super-resolution (Li *et al.* 2022, Luo *et al.* 2023). Unlike discriminative models, generative models model the joint probability distribution between the data and the labels. At the same time, DDPM has demonstrated an advantage over other generative models, like the generative adversarial network and energy-based models (Ho *et al.* 2020), due to its advanced design, like the Markov chain that simulates the diffusion process and the marginal Gaussian noise removal. Consequently, the success of DDPM makes it a great candidate for the focus restoration task and a promising tool that advances HT imaging.

We propose PostFocus, an efficient approach to improve the yield and accuracy of data from HT phase contrast microscopy, post-acquisition. Specifically, we screened the OOF images using state-of-the-art image classification models and leveraged multiple focus restoration models to restore image focus. The combination of the two modules allows an efficient and adaptive image restoration workflow. Through comprehensive quantification, we illustrate the best models and how they improve image analysis results and the yield of high-quality data.

## 2 Materials and methods

### 2.1 Image dataset acquisition

We recorded images of cells or beads within polydimethylsiloxane (PDMS) nanowell arrays fabricated following published protocols (Liadi *et al.* 2013, 2015) to collect in-focus and OOF images. The experiments, the datasets, and the imaging setup have been described in detail (Lu *et al.* 2019, Montalvo *et al.* 2024). For collecting datasets with multiple focal planes, we imaged the cells within the nanowells by utilizing three different *z*-planes: the optimal focal plane as determined by an expert observer, and two OOF *z*-planes shifted 10 µm in either direction from the in-focus plane (imaged within 100 ms, cell movement is therefore negligible). Specifically, TIMING nanowells are 50 µm deep, and the optimal imaging zone is ∼20 µm from the bottom of the wells. Moreover, an offset greater than 10 µm can make cells disappear and leave no features for restoration. Hence, the shift of 10 µm is an empirical boundary condition that introduces significant morphological change without making the restoration task infeasible.

After applying the image preprocessing tools described previously (Liadi *et al.* 2015, Lu *et al.* 2019), we obtained pairs of in-focus and OOF (randomly picked between two OOF planes) nanowell images for SKOV3, NALM6, and beads (1654, 1299, and 1350 nanowells, respectively), with randomly added pairs of identical in-focus images into the dataset (consisting of 25% of data). As shown in visual examples (Fig. 1B), the cells in OOF images lacked critical features and led to incorrect segmentations, contact detections, and ultimately more inaccurate analysis results.

### 2.2 Focus assessment through image classification

To identify the OOF images for restoration, we generated a detection model for automated focus assessment after image preprocessing (Lu *et al.* 2019). We trained multiple five well-studied (Nawaz *et al.* 2018, Zhang *et al.* 2019b, Wang *et al.* 2020, Sarwinda *et al.* 2021, Zhou *et al.* 2022) off-the-shelf deep neural networks on a binary classification task, including AlexNet (Krizhevsky *et al.* 2012), ResNet50 (He *et al.* 2016), GoogLeNet (Szegedy *et al.* 2016), ViT B-32 (Dosovitskiy *et al.* 2020), and CoAt-Net (Dai *et al.* 2021). We trained the models with an image dataset with 4303 randomly picked images (OOF: IF = 21:20) and took 75% of them for a 5-fold cross-validation experiment.

We trained the models from scratch and then compared their sizes and classification performance to find the best one for our workflow. We used validation accuracy as the primary matrix to quantify and compare the models’ performance in cross-validation experiments, for which we chose a batch size of 50 and trained the model for 100 iterations. During training, we computed the cross-entropy loss (Mao *et al.* 2023) as the gradient for the Adam optimizer (Kingma and Ba 2014), which updated the model’s weights with a learning rate of 10^−4^ and a L2 weight decay (Phaisangittisagul 2016) coefficient of 10^−5^. In addition, we normalized the image data w.r.t. ImageNet dataset (Krizhevsky *et al.* 2012), then applied rotation, contrast adjustment, and brightness adjustment at random to augment training data.

### 2.3 Image focus restoration using deep learning models

We evaluated three deep neural networks to find the best focus restoration model. We considered the Deep Multi-Patch Hierarchical Network (DMPHN, Zhang *et al.* 2019a), Uformer (Wang *et al.* 2022), and de-noising DDPM (Ho *et al.* 2020) for our focus restoration task. We followed the original three-level hierarchical design for DMPHN and the original four-layer architecture of window-based attention blocks for Uformer. For DDPM, we followed the design in previous works (Ho *et al.* 2020, Wolleb *et al.* 2022), including a 13-layer, U-shaped architecture of convolution and attention blocks and a 1000-step Gaussian noise addition schedule (variance starts from 10^−4^ and ends at 0.05).

We trained the three models using the in-focus and OOF image pairs to compare their performance. We trained the three models from scratch using the Adam optimizer with the Charbonnier loss (Gajera *et al.* 2021) for DMPHN and Uformer and the mean-squared error (MSE) loss for DDPM, and the rest of the hyperparameters of the optimizer are the same as training the OOF image classifier. Since the SKOV3 cells have the most complicated geometry and motile nature, we used the SKOV3 dataset to compare the models’ performance. After training, we evaluated the best weights using four quantitative matrices against the validation set (with a 4:1 train/validation split). Moreover, considering the randomness involved when the DDPM generates output, we generated five output images from each OOF input and took an average of them for evaluation.

To evaluate the model’s performance, we picked four quantitative matrices to assess the quality of the output images and the fidelity of the restored content. On one hand, we use two matrices to quantify the quality of the generated images. The Tenenbaum gradient (Sun *et al.* 2004) indicates the sharpness of the entire image, and the peak signal-to-noise ratio (PSNR; Hore and Ziou 2010) quantifies the quality of reconstructed images. On the other hand, we computed the Pearson correlation coefficient (PCC, $\frac{\sum \left(J_{i}-\bar{J}\right)\left(K_{i}-\bar{K}\right)}{\sqrt{\sum {\left(J_{i}-J\right)}^{2}\times \sum {\left(K_{i}-\bar{K}\right)}^{2}}}$, *J* and *K* are two input images) to measure the accuracy of the restored content. We computed PCC in two ways: considering all pixels in the image or those detected as edges defined by the Canny filter (McIlhagga 2011). Lastly, to quantify the quality of the overall distribution of generated images, we also computed the Fréchet inception distance (FID; Heusel *et al.* 2017) between generated and ground truth images.

### 2.4 Analysis of the robustness, credibility, and usefulness of the focus restoration module

We conducted three tests to evaluate the model’s robustness. First, we trained the model with the full dataset with all images (with two types of cells and fluorescent beads) and tested with the same quantitative matrices. This will illustrate how well the model copes with the data variability. Since objects change their morphology in different patterns when being OOF, we expect deep learning models to learn and apply various blur kernels correctly.

Moreover, we evaluated if the generative model affects data credibility through hallucination. Specifically, hallucination refers to the generation of misleading or false responses by artificial intelligence, which has attracted increasing attention as new models become powerful (Salvagno *et al.* 2023, Zhang *et al.* 2023). Hence, we imaged the NALM6 cells at one additional plane 5 µm away from the in-focus plane as the second set of OOF images. By comparing DDPM trained with different OOF images, we demonstrated how DDPM generates details and the trustworthiness of these images.

Lastly, we used the TIMING cell segmentation module (Lu *et al.* 2019) to segment the cells in image sequences. The segmentation module leverages a MaskRCNN model (He *et al.* 2017) trained with ImageNet (pretraining) and 8804 manually annotated TIMING images of NALM6 and T cells (with 17 602 cells). The model reached an average IoU of 0.87 ± 0.07 on the testing dataset of 2265 images (4550 cells). The testing image sequences contain NALM6 cells under three conditions: in-focus, OOF, and restored by restoration module. Next, we defined the regions with overlapping masks as cell–cell contact regions, computed the PCC and MSE of the contact region masks of OOF, and restored images. In addition, we tracked the number of cells detected and computed the error. All tests use the results on in-focus videos as ground truth. These tests will demonstrate how the focus restoration improves the image analysis quality and, thus, its importance in imaging-based assays.

## 3 Results

### 3.1 Deep neural networks reliably identified OOF images

**Figure 2.**
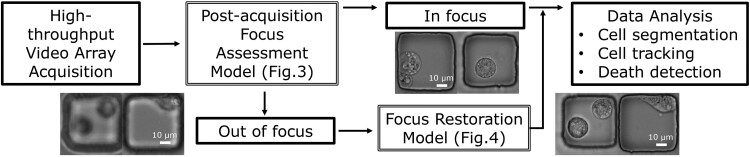
Proposed workflow (PostFocus) to identify images that are OOF and selectively apply focus restoration to only the OOF images. This efficiently improves the overall yield by reducing cell detection and tracking errors caused by loss of focus and degraded image quality. Figures 3 and 4 discuss the individual modules in detail.

**Figure 3.**
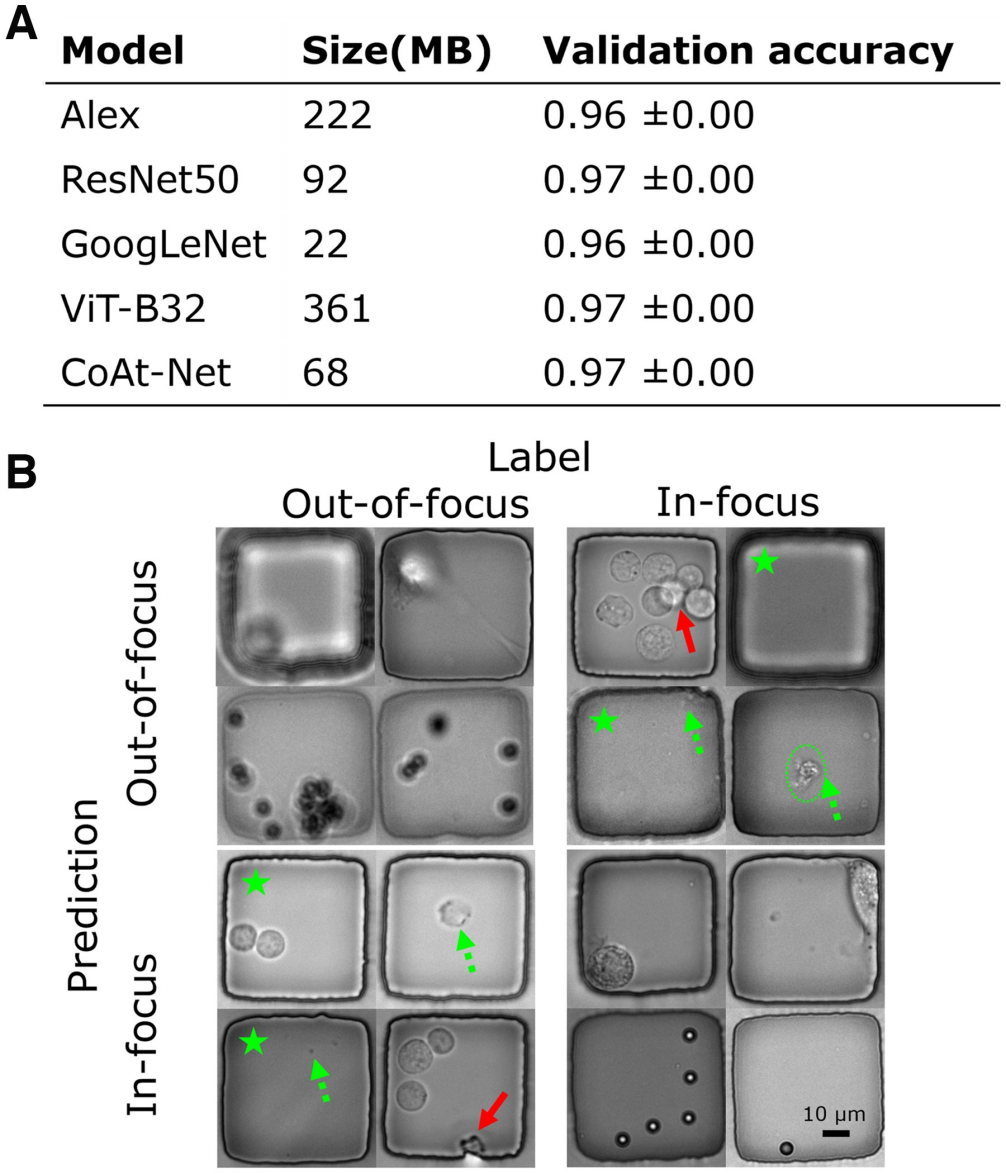
Off-the-shelf deep neural networks showed reliable performance in identifying OOF images. (A) Table summarizing the performance of five widely used models. (B) Visual examples of CoAt-Net’s prediction in the format of a confusion matrix showed that despite the overwhelmingly successful performance, there were still rare failures due to errors from automated annotation and the presence of debris (the first and third quadrants).

To efficiently restore image focus, we developed PostFocus, a two-step data processing workflow (Fig. 2) leveraging two different neural networks. To save the computational cost for restoration, the first part is an image classifier conducting the focus assessment after image acquisition and preprocessing. Hence, we first investigated the capability of deep neural networks to identify OOF images, and our results showed the robustness of all five models (Fig. 3A, accuracy >96%) comparable to the focus estimation results for different image modalities (Xue *et al.* 2022).

All the models we tested achieved high classification accuracy (Fig. 3A), indicating that evident features associated with focus determination exist in our data. To test the importance of normalization, we conducted the same experiment without the normalization process and obtained similar results (Supplementary Table S1). These results confirmed that our data preprocessing and normalization process successfully preserved relevant features. Instead of learning less relevant features and underperforming (Qadir *et al.* 2019), AlexNet performed similarly to deeper models. Moreover, despite learning long-range feature dependencies with the attention mechanism (Vaswani *et al.* 2017), there was only a 1% difference in accuracy between ViT or CoAtNet and the other models. Since all models detected critical features of OOF images, we chose CoAtNet for our workflow due to its small size (68 MB) and high accuracy.

Next, we investigated the source of error from CoAtNet by visualizing sample predictions in the format of a confusion matrix (Fig. 3B). The major sources of error identified from Fig. 3B are cells located at different axial positions (dark solid arrows), debris (bright dashed arrows and circle), and rare wrong labels (stars) from automatic annotation pipeline. These factors led to noisy or partially OOF images with a low frequency in our dataset. Despite these rare errors, the CoAtNet classifier is still reliable as it performed well quantitatively and qualitatively (the top left and bottom right corner of Fig. 3B).

Next, we used the trained CoAtNet to quantify the scale of OOF issue in general TIMING datasets (Fig. 1C). We measured the frequency of OOF frames of each video and plotted the number of OOF (OOF frame frequency >5%) and in-focus videos of seven TIMING datasets (the first four datasets are derived from TIMING imaging T cell killing experiments against the SKOV3 cells; and the last three datasets are from T cell killing experiments against NALM6 cells, Montalvo *et al.* 2024). As shown in the bar graph, there are (1266–2708) videos in each dataset, and OOF frequency was (6%–15%) for NALM6 datasets and (14%–96%) for SKOV3 datasets. These data demonstrate the scale of the issue and illustrate that OOF blur poses a severe issue for HT phase contrast microscopy.

### 3.2 DDPM demonstrated the best focus restoration performance

Following the OOF image identification, the next part in PostFocus (Fig. 2) is the focus restoration module for minimizing the errors in subsequent image analysis. In this section, we compared three models: two discriminative models; CNN-based DMPHN (Zhang *et al.* 2019a) and transformer-based Uformer (Wang *et al.* 2022), and DDPM (Ho *et al.* 2020), a generative model with a hybrid (convolution and attention) architecture and diffusion operation.

**Figure 4.**
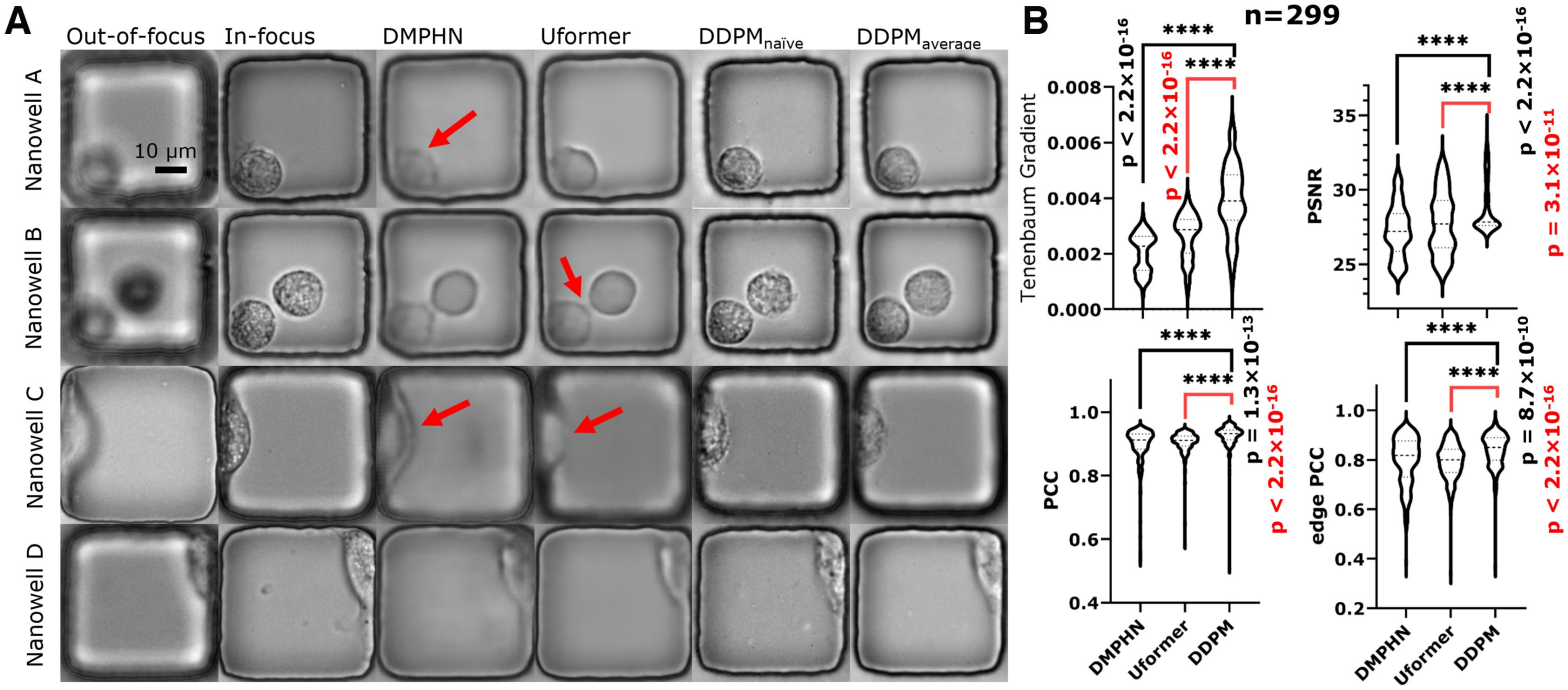
The proposed diffusion model (DDPM) performed the best on focus restoration both qualitatively and quantitatively. (A) Visualization of the performance on the different models on four sample nanowells. DDPM was able to generate cell images with fine morphological details and complete boundaries. On the other hand, discriminative models (DMPHN and Uformer) generated cells with insufficient contrast, connecting, or even crumbling boundaries (arrows). (B) We evaluated the images restored by the three models using the Tenenbaum gradient, peak PSNR, and PCC. Quantitatively, DDPM outperformed the other models with the highest distribution of Tenenbaum Gradient, PSNR, and PCC values.

Sample images processed by three models demonstrated that DDPM performed the best. DDPM produced images with the best cell contrast and boundary completeness. For example, the noise in the background (Fig. 4A, Nanowell A) made DMPHN generate a cell with low contrast (arrow), potentially causing segmentation errors. DDPM, on the other hand, remains robust against noise as it generates output by removing noise. Similarly, background noise between cells can lead to unwanted artifacts. Nanowell B in Fig. 4A showed that discriminative models generated connected cells (arrows) due to such noise, while DDPM again generated clear, separated cells. In addition, the superiority of DDPM is evident when the cell is attached to the wall (Fig. 4A, Nanowell C and D). The restoration of these images is difficult as cells tend to elongate when adhered to the wall, increasing the complexity of the geometry and morphology. Despite these challenges, DDPM generated cells with complete boundaries, whereas discriminative models failed (arrows). These samples illustrate that DDPM generates cells with the best boundary quality.

In addition to qualitative analysis, we leveraged quantitative matrices to verify DDPM’s performance. The distribution of the Tenenbaum gradient (Fig. 4B) demonstrated that DDPM generates the sharpest images. This is because DDPM is the only model that can generate morphological details, while discriminative models failed to learn the mapping between OOF images and morphological details. Moreover, DDPM generated complete boundaries contributing to a higher distribution of edge-PCC and reaching the best FID score (Supplementary Table S2), which indicates the best similarity to ground truth. In summary, despite discordance in cellular details within the cell boundary, our quantitative analysis illustrated that DDPM is the best model for restoring accurate and sharp cell boundaries.

**Figure 5.**
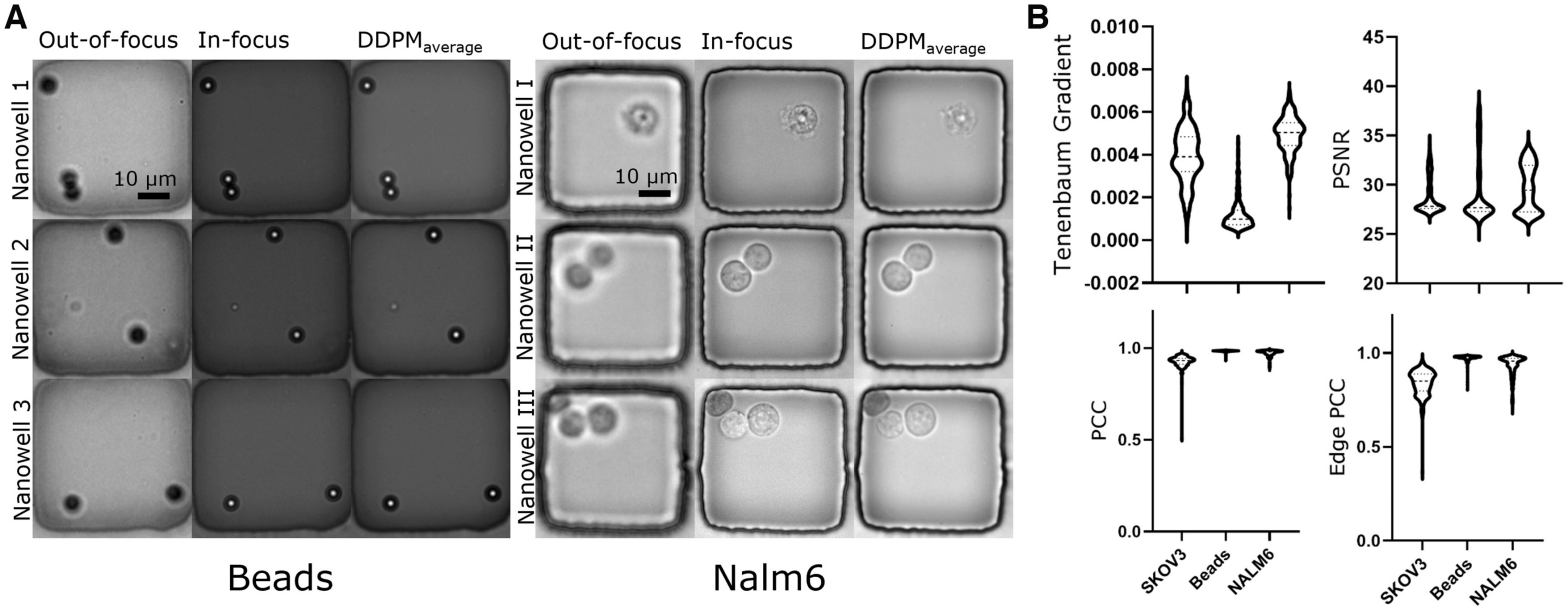
The diffusion model (DDPM) demonstrated consistent performance across multiple cell types and beads. (A) Sample images of restoring OOF images of beads and NALM6 tumor cells with the DDPM model. (B) Quantitative comparison of DDPM’s restoration performance on different objects. The model performed best for the beads (best peak PSNR and PCC; we discussed the distribution of Tenenbaum gradient in detail in Section 3.2) due to their simple morphology (Panel A).

Next, we verified DDPM’s generalizability by training and testing it with datasets with different types of cells (NALM6 and SKOV3) and beads. DDPM remained robust as the sample images showed (Fig. 5A). This performance also shows that the DDPM model perceives the morphological difference between cells and beads to generate authentic output. In addition, we provide the quantitative analysis (Fig. 5B) of DDPM’s performance. The PCC and edge PCC distributions showed a significant difference between SKOV3 and the other object types. This is because NALM6 cells and beads are usually circular objects, whereas SKOV3 cells elongate and change shape frequently (Fig. 4A, Nanowell C and D), making it challenging to delineate complex boundaries. On the other hand, since the beads have the most straightforward morphology, DDPM generated the least amount of details, leading to the lowest distribution of the Tenenbaum gradient. In short, our results demonstrated that DDPM is a robust model that can cope with restoring multiple types of objects.

In summary, DDPM successfully restored multiple types of objects and outperformed other models in restoring solid tumor cells (SKOV3 cells). Our results demonstrated that learning the distribution of in-focus image data through the diffusion process is beneficial while the learned inverse function of PSF can lead to unsatisfactory cell boundary quality. Therefore, we consider DDPM the best option for our HT focus restoration module.

**Figure 6.**
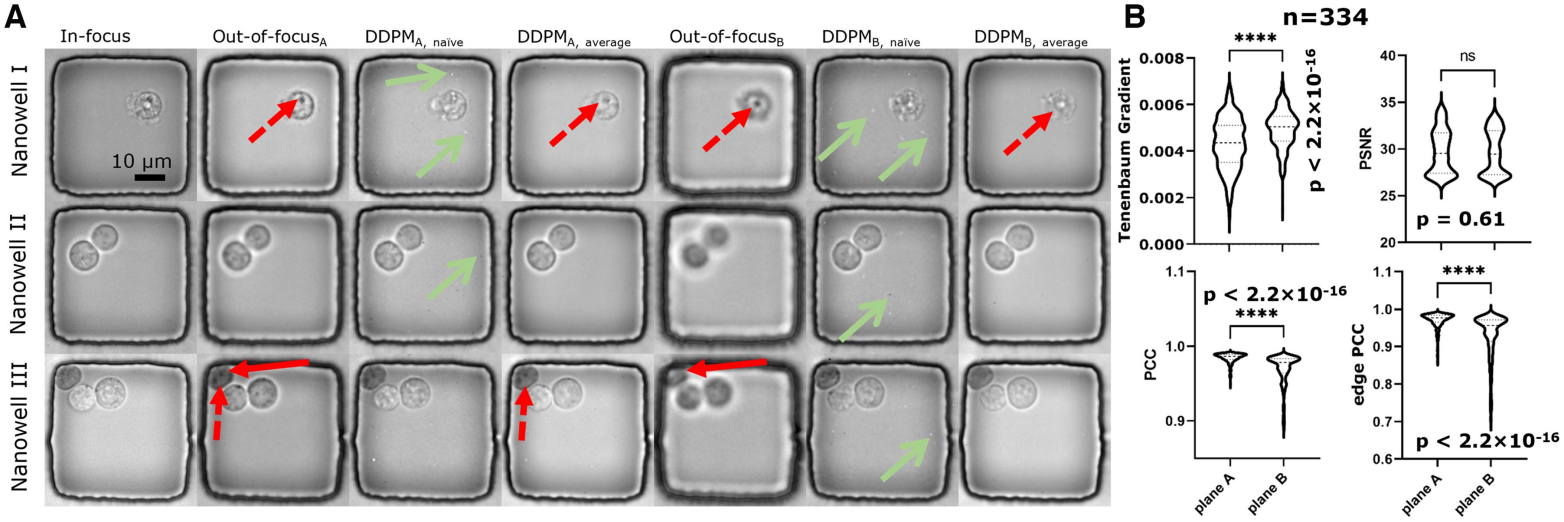
Averaging the output from DDPM improves the reliability of the generated image. (A) Visual examples of the relationship between the generated morphological details and input images. We used images taken at two planes (a and b, 5 µm apart) as the OOF priors to train the DDPM model. After taking the average for five generated images, the remaining morphological details generated have visual counterparts in the prompt (pairs of dashed arrows), making our approach based on the DDPM reliable and free from hallucination. (B) DDPM achieved better quantitative performance using OOF priors from the first plane (better distribution of PCC despite the lower Tenenbaum gradient as discussed in Section 3.3). This is because the first plane is closer to the in-focus plane and thus contains morphological detail resembling ground truth images (solid, close-ended arrows in panel A).

Moreover, we analyzed DDPM’s output quantitatively using different priors to show the model’s trustworthiness (Fig. 6B). First, we obtained sharper images from plane B with a higher distribution of the Tenenbaum gradient. Still, the overall image quality is similar for the two planes as the PSNR distribution suggested. The difference in image sharpness can originate from the level of details in the prior. Since plane B possesses less information, the variance between random image generations became lower and led to a sharper image after taking an average over a more consistent set of images. On the other hand, DDPM’s output achieved a better PCC score distribution when using plane A for priors. Since plane A is closer to the in-focus plane (5 against 10 µm), images taken there contain more information and better resemble in-focus ground truth. For example, the cell at the top left corner of Nanowell III (Fig. 6A) appears to be of different shape in planes A and B (solid, close-ended arrows), and the former has the geometry closer to the ground truth, making the generated images resemble ground truth better. Hence, the generation based on prior set A achieved better PCC scores, verifying that image generation is a robust process regulated by the prior.

Based on our qualitative and quantitative results, we validated the robustness and reliability of DDPM. To minimize the discordance in cellular details we had observed, we hypothesized that we can improve reliability by generating and averaging multiple outputs. Our results validated this hypothesis and demonstrated that averaging improved the robustness of DDPM and minimized concerns of DDPM’s hallucinations.

### 3.3 The focus restoration improved the accuracy of cell detection and cell–cell contact detection

After validating DDPM’s performance and reliability for focus restoration, we conducted cell detection and contact detection on image sequences with different focal conditions to demonstrate how our workflow can benefit timelapse microscopy. It is necessary to quantify cell–cell interactions as they play critical roles in tumor-killing mechanisms (Adams and Hamilton 2019, Espie and Donnadieu 2022) of immune cells. For example, the detection of effector-target cell contact regions indicates the formation of the immune synapse and, therefore, the target cell killing process (Jang *et al.* 2015, Im *et al.* 2020, Chockley *et al.* 2023).

**Figure 7.**
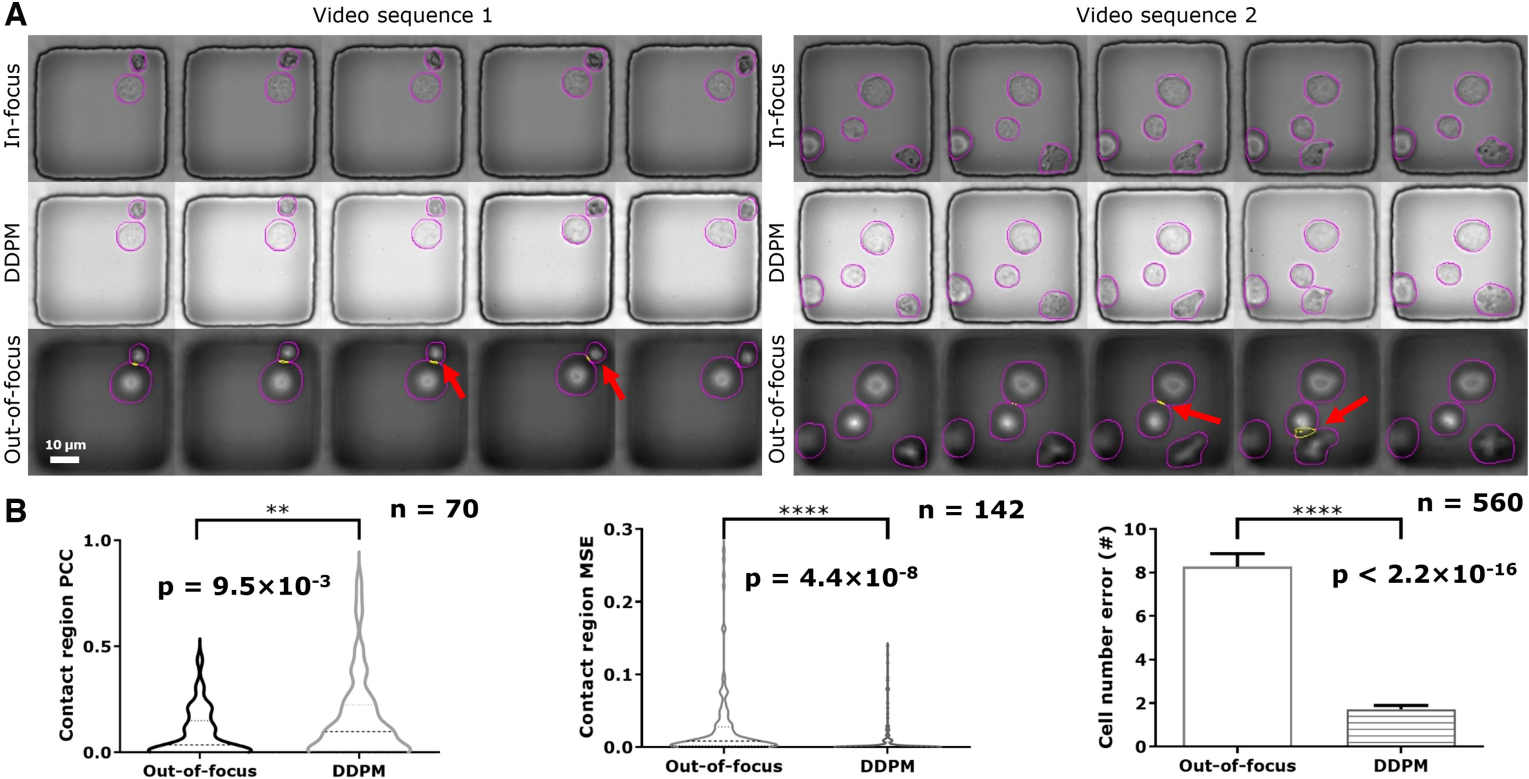
Focus restoration improved the accuracy of cell detection and delineation of cell–cell contacts. (A) Two sets of sample videos illustrating that OOF images lead to errors in cell detection and contact. We present in-focus ground truth images at the top row, DDPM-restored images in the middle row, and OOF images at the bottom. The darker contour denotes cell boundary, and the brighter color represents the contact region. When images are out of focus, the blurry boundary will lead to errors in cell detection and, thus, in the detection of contact (arrows). (B) Quantitative profiling of focus restoration improving contact and cell detection. The contact detection yielded a higher PCC and lower MSE for the restored image sequences against the in-focus ground truth. In addition, the cell detection module made significantly fewer errors after focus restoration.

We provide two sample video sequences (Fig. 7A) to illustrate how focus restoration improved performance on the two tasks. Compared with in-focus cells, the OOF cells usually have thicker cell boundaries and take up more area in images, leading to a higher chance of false positive detection of contact. For example, the cells in the in-focus version of both video sequences are not in contact according to segmentation results. However, the same model drew larger masks to encircle the blurred cells, making the masks intersect and indicate a false contact event (yellow and arrows in Fig. 7A). In comparison, images after focus restoration (row DDPM, Fig. 7A) have sharp cell boundaries, and the segmentation model made no false contact detection. In short, re-focusing the images provided clear object boundaries and improved the segmentation accuracy.

In addition, focus restoration also reduced cell detection errors by recovering visual features for OOF objects. Following the cell boundary, the cell body will also become blurry as the axial displacement from the optimal plane of a cell increases. As shown in the sample video (Supplementary Video S2, 20th frame), the OOF cell became blurry, leading to under-segmentation. Fortunately, after focus restoration, the cell detection model provides accurate results.

To support our claims, we computed the PCC and MSE of the contact area detected by the segmentation module with results from in-focus videos as reference. The results demonstrated a noticeable improvement brought by focus restoration. With restoration based on DDPM, the mean PCC increased from 0.1 ± 0.1 to 0.2 ± 0.2 (Fig. 7B, left), and the mean MSE decreased from 0.02 ± 0.005 to 0.01 ± 0.002 (Fig. 7B, middle). In addition, the segmentation model reached a higher accuracy in predicting the number of cells with focus restoration, from a mean value of 8 ± 14 to 2 ± 4 cells (Fig. 7B, right). Our qualitative and quantitative results verified how focus restoration improved image quality and, therefore, better image analysis accuracy.

## 4 Discussion and conclusion

HT imaging and deep learning-based computer vision algorithms are essential tools for profiling functional cell biology (Moen *et al.* 2019, Evans III *et al.* 2022, Serrano *et al.* 2023). To ensure the best analysis accuracy, the strategy to minimize blurry images has long been a topic of interest as modern algorithms are sensitive to the change in textural details by blur kernel, leading to errors in cell classification and cell segmentation (Chen *et al.* 2021, Wang and Han 2022). Our label-free approach can solve the issue without potential drawbacks like photo-toxicity or photo-bleaching (Oh *et al.* 1999, Vicente *et al.* 2007).

In this work, we propose PostFocus, an efficient two-step pipeline that leverages a state-of-the-art image classification model and the DDPM to selectively restore OOF images, with protection against hallucination through averaging. PostFocus is a practical solution to the common challenge of automated image analysis against intermitted focus loss in HT time-lapse bio-imaging without modifying the image acquisition instrumentation/process. Nevertheless, as we study different biological systems, we expect out-of-distribution morphology that impacts the model’s performance and leads to new artifacts from image generation. Hence, a constant assessment, update, and validation of the restoration model is necessary. Although DDPM has demonstrated superior performance in focus restoration, its nature makes it computationally more expensive (Supplementary Table S3). Since it is computationally expensive, as stated above, we have employed a two-step pipeline to ensure that focus restoration is only performed when necessary. In the future, we look forward to further improvements to speed by allocating high-performance computing resources and enhancing efficiency through methods like the de-noising diffusion implicit model (Song *et al.* 2020).

Moreover, the nature of focus restoration makes it unlikely for any algorithms to restore morphological details completely. Although real-time focus tracking is the best approach to preserve biological complexity during acquisition, it is not always practically feasible. For example, for HT time-lapse imaging assays, the faster acquisition forces tradeoffs on the time allocated to the acquisition of each individual image. In addition, for live cell imaging assays, imaging multiple planes to acquire the best in-focus image can also lead to extended exposure of cells to light, therefore altering cell biology and accelerating cell death. In summary, while real-time focus tracking is not always feasible, computational tools that offer the ability to restore focus after acquisition represent an advance to HT time-lapse imaging of live cells.

**Figure 8.**
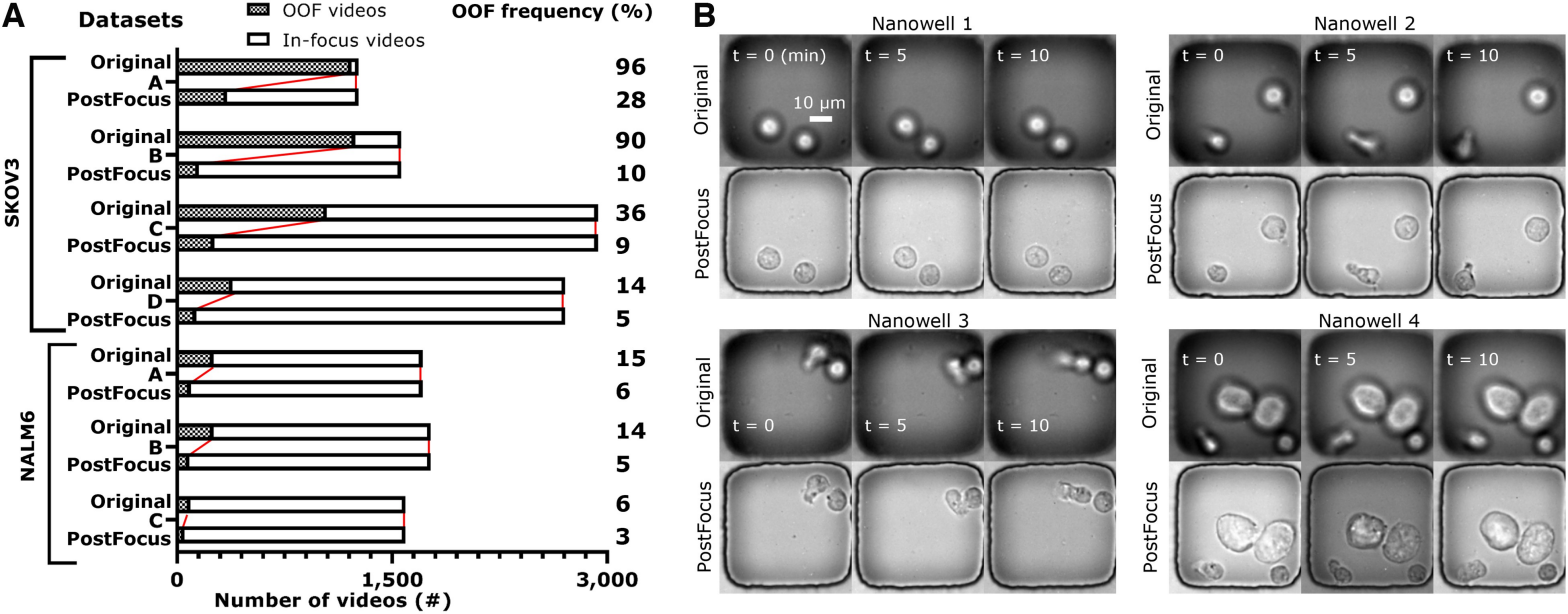
The proposed focus restoration pipeline recovered 75% of all the OOF time-lapse videos of nanowell from multiple TIMING datasets. (A) Bar graph of seven separate TIMING datasets (same ones in Fig. 1C) before (top bars) and after (bottom bars) applying our focus restoration pipeline. The shaded region (left of each bar) indicates the number of videos with more than 5% of OOF frames, the blank region (right) stands for the number of in-focus videos. The change in colors and the number next to each pair of bars indicate the significant OOF frequency change. (B) Four sample videos from SKOV3 datasets A and B. The comparison between the original (top row) and PostFocus (bottom row) versions demonstrates the improvement in image quality.

In addition to improving image analyses, we provided evidence of PostFocus’s effectiveness in recovering nanowell videos for the TIMING assay. When applied to the seven TIMING datasets discussed in Section 3.1, PostFocus reduced the OOF frequency from 96%, 80%, 36%, 14%, 15%, 14%, and 6% to 28%, 10%, 9%, 5%, 6%, 5%, and 3%, respectively (Fig. 8A). For all datasets, PostFocus recovered a total of 75% of OOF videos (Fig. 8B provides samples). Hence, PostFocus can advance the TIMING assay by reducing the frequency of erroneous readouts due to OOF blur and improving the yield of fully usable nanowell videos from expensive experiments. Moreover, the decrease in data throughput due to OOF is not an issue exclusive to TIMING assays, and the exclusion of OOF images is still necessary for automated image analysis accuracy. For example, microfluidic devices have shown usefulness in automating microscopy for high-resolution cell imaging (Tam *et al.* 2014). However, cells overlapping or exhibiting dynamic behavior can still lead to OOF blur. On the other hand, as 3D imaging enables high-resolution cell analysis (Dekkers *et al.* 2023) and 3D scaffolds unveil diverse cell behaviors (Parodi *et al.* 2020), restoring image focus will be beneficial for analyzing these data. With proper training for domain adaptation, we anticipate that PostFocus can become a valuable tool for a broader community of microscopy users.

On a broader level, generative models have revolutionized the field of computer vision by unprecedented output quality and strong learning ability. Such innovation in vision algorithms has provided exciting possibilities to advance HT microscopy without the need for hardware upgrades. For instance, by learning the complicated mapping between data, diffusion models achieved excellent performance on complex image editing tasks like object addition/removal and image in-painting (Avrahami *et al.* 2022, Saharia *et al.* 2022a). These capabilities enable image curation (Barrett *et al.* 2021) and post-acquisition restoration of lost content due to randomly introduced extrinsic defects (Wang *et al.* 2019a, Ma *et al.* 2021). In addition, the instrumentation limitation will always create a tradeoff between spatial and temporal resolution, limiting the yield of data. Thankfully, recent works have successfully overcome these tasks through super-resolution (Van and Preza 2021, Kawar *et al.* 2022, Saharia *et al.* 2022b) and frame interpolation (Höppe *et al.* 2022, Danier *et al.* 2023). Hence, it is now possible to profile at high spatial and temporal resolution (Qiao *et al.* 2023, Priessner *et al.* 2024). Lastly, for discriminative tasks, diffusion models also demonstrated exceptional performance and outperformed traditional models in tasks like image classification and image segmentation (Zimmermann *et al.* 2021, Wolleb *et al.* 2022). Overall, we anticipate that the proposed method and future applications of generative models can become valuable image-processing modules in diverse quantitative time-lapse microscopy workflows, ultimately facilitating sensational discoveries in various fields.

## Supplementary Material

btae467_Supplementary_Data
