# Supplementary figures and images for: PostFocus: automated selective post-acquisition high-throughput focus restoration using diffusion model for label-free time-lapse microscopy
Source: Bioinformatics. 2024 Jul 23;40(8):btae467. doi: 10.1093/bioinformatics/btae467 (PMC11520405; doi:10.1093/bioinformatics/btae467)

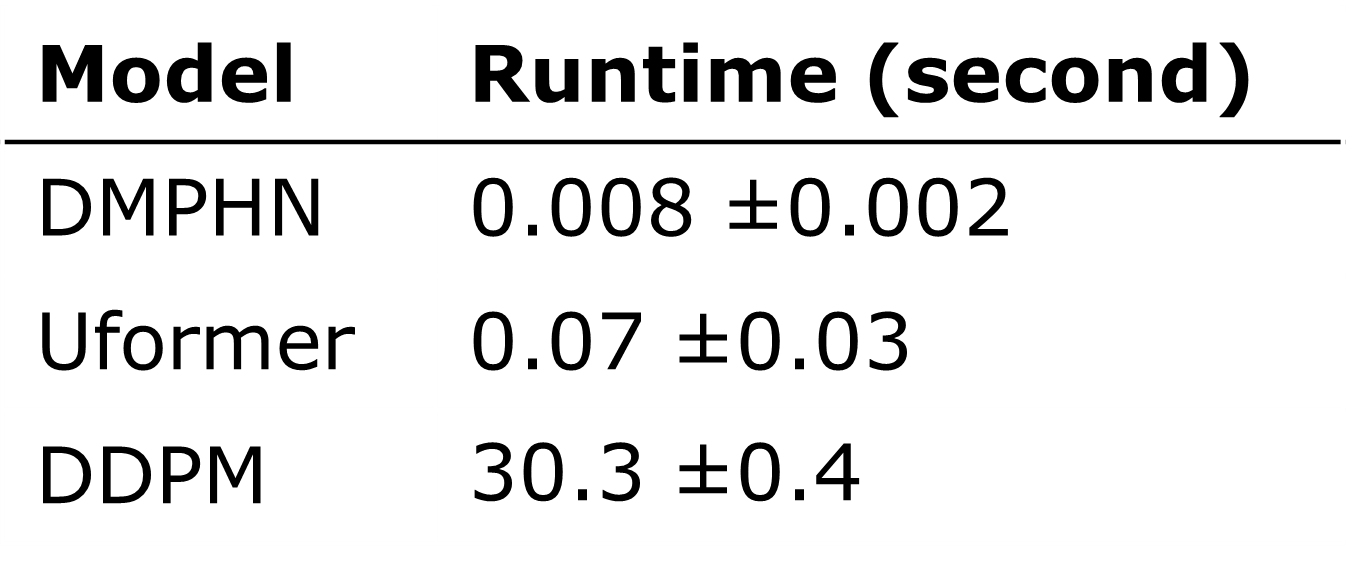

Supplement: btae467_Supplementary_Data [file btae467_supplementary_data.zip › PostFocus_Supplementary Table 3. Runtime of the three focus restoration models.tif]

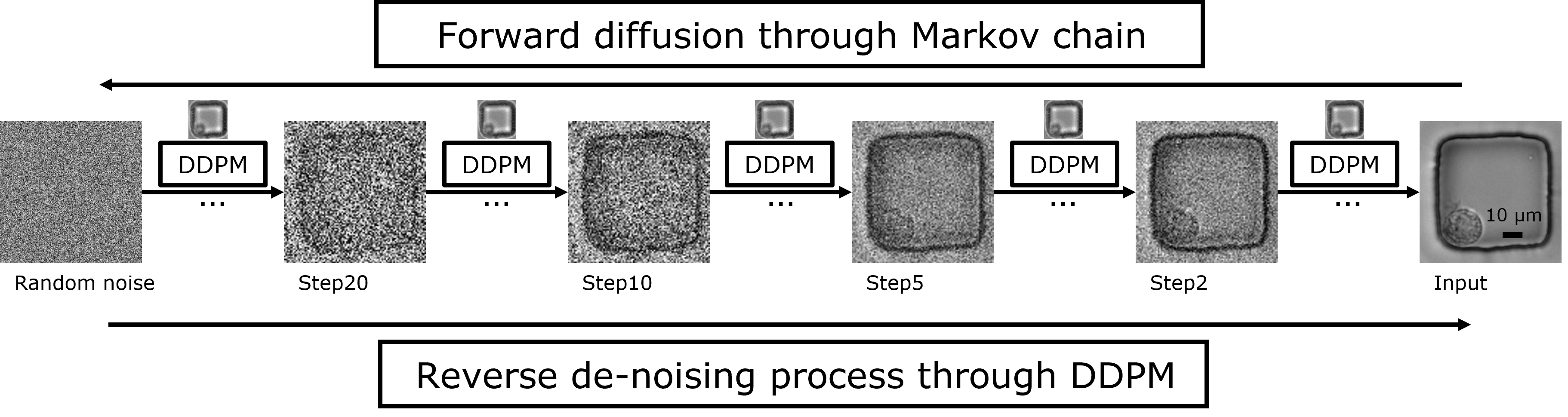

Supplement: btae467_Supplementary_Data [file btae467_supplementary_data.zip › PostFocus_Supplementary Figure 1. Schematic illustration of the working mechanism of DDPM.tif]
